# Diagnostic Utility of Upper Airway Ultrasonography in Adults with Suspected Obstructive Sleep Apnea: A Systematic Review

**DOI:** 10.3390/jcm15103720

**Published:** 2026-05-12

**Authors:** Anutta Terawatpothong, Hitoshi Hotokezaka, Noriaki Yoshida, Irin Sirisoontorn

**Affiliations:** 1 Division of Orthodontics, Department of Preventive Dentistry, International College of Dentistry, Walailak University, 87 Ranong 2 Road, Dusit, Bangkok 10300, Thailand; 2 Department of Orthodontics and Dentofacial Orthopedics, Nagasaki University Graduate School of Biomedical Sciences, 1-7-1 Sakamoto, Nagasaki 852-8588, Japan

**Keywords:** obstructive sleep apnea, upper airway, airway collapsibility, diagnostic performance, polysomnography, ultrasonography

## Abstract

**Background:** Ultrasonographic assessment of the upper airway has emerged as a non-invasive method for evaluating obstructive sleep apnea (OSA), offering advantages including wide accessibility and absence of ionizing radiation. However, the diagnostic validity and standardized screening thresholds for ultrasonographic parameters remain unclear. **Methods**: A systematic literature search was conducted in PubMed, Scopus, and ScienceDirect from database inception to February 2026. Eligible studies enrolled adults with suspected OSA, used in-laboratory polysomnography (PSG) as the reference standard, and assessed upper-airway structures using ultrasonography. Studies reporting diagnostic performance metrics (sensitivity, specificity, AUC, or diagnostic thresholds) or quantitative associations with apnea–hypopnea index (AHI) were included. Risk of bias was assessed using QUADAS-2. Owing to methodological heterogeneity, findings were synthesized qualitatively. **Results**: Six studies (n = 473 participants) met the inclusion criteria. Evaluated parameters included tongue base thickness, lingual artery distance, lateral pharyngeal wall thickness, dynamic airway dimensional changes, and tongue stiffness. Three studies reported threshold-based diagnostic performance, although only one provided a complete diagnostic contingency table. Dynamic retropalatal percentage change demonstrated the highest diagnostic performance (AUC up to 0.989; sensitivity 97%; specificity 93.3%). Other studies demonstrated significant morphologic associations with OSA severity but lacked externally validated diagnostic thresholds. **Conclusions**: Ultrasonographic upper-airway assessment demonstrates promising structural and functional correlates of OSA. However, robust diagnostic accuracy evidence and standardized thresholds remain limited. Further prospective studies with standardized acquisition protocols and predefined diagnostic thresholds are required before ultrasound can be incorporated into routine OSA screening pathways.

## 1. Introduction

Obstructive sleep apnea (OSA) is a prevalent sleep-related breathing disorder characterized by recurrent upper-airway collapse during sleep, resulting in intermittent hypoxemia, sleep fragmentation, and substantial cardiometabolic and neurocognitive morbidity [1,2]. Global epidemiological estimates suggest that hundreds of millions of adults worldwide may have at least moderate OSA (apnea–hypopnea index (AHI) ≥ 15 events/hour), a threshold frequently associated with increased cardiovascular risk and treatment indication [1]. Despite its high prevalence and substantial clinical burden, OSA remains substantially underdiagnosed, largely due to the limited accessibility, cost, and logistical demands of in-laboratory polysomnography (PSG), the current gold standard for diagnosis [3].

Upper-airway anatomy and craniofacial morphology are recognized determinants of airway collapsibility and OSA pathophysiology [2]. Mandibular retrusion, maxillary transverse deficiency, vertical facial growth patterns, and soft tissue enlargement are recognized contributors to structural vulnerability of the upper airway. Consequently, OSA has increasing relevance within orthodontics and dentofacial orthopedics, where mandibular advancement devices, maxillomandibular advancement, and other skeletal interventions can directly influence upper-airway anatomy and function [4]. However, most orthodontic assessment relies primarily on skeletal imaging modalities, while accessible chairside tools for evaluating dynamic soft tissue airway behavior remain limited.

Ultrasonography has emerged as a non-invasive, radiation-free imaging modality capable of evaluating upper-airway soft tissue structures and dynamic airway behavior at the point of care. A previous systematic review and meta-analysis evaluated surface ultrasound parameters for OSA screening, incorporating both airway and non-airway measurements in perioperative and general populations [5]. More recently, a quantitative synthesis pooled ultrasonographic structural differences between OSA and non-OSA groups [6]. Although these reviews provided valuable descriptive and comparative insights, they incorporated heterogeneous parameters, combined airway and non-airway structures, and did not specifically focus on predefined diagnostic thresholds within a strict diagnostic accuracy framework.

Importantly, correlations with AHI or differences in group means do not necessarily translate into clinically actionable screening criteria. Several individual studies have reported promising diagnostic parameters, including the distance between lingual arteries [7], dynamic retropalatal percentage change [8], deep-breath–related airway dynamics [9], tongue stiffness assessed by shear-wave elastography [10], dynamic tongue base thickness [11], and lateral neck soft tissue measurements [12]. Nevertheless, substantial heterogeneity exists in acquisition protocols, AHI thresholds, and the reporting of diagnostic performance metrics, including sensitivity, specificity, receiver operating characteristic (ROC) analysis, and area under the receiver operating characteristic curve (AUC), which summarizes overall diagnostic discrimination.

Furthermore, methodological concerns such as spectrum bias and case–control design have been shown to inflate diagnostic performance estimates in test accuracy research [13]. Within clinical pathways, ultrasound must be interpreted relative to established diagnostic standards such as PSG [3] and validated screening instruments including STOP-Bang questionnaires [14]. Accordingly, a focused synthesis of ultrasonographic diagnostic performance restricted to upper-airway structures and PSG–confirmed OSA remains warranted.

Therefore, this systematic review aimed to critically evaluate the diagnostic performance and morphologic associations of ultrasonographic upper airway parameters in adults with suspected OSA, using PSG as the reference standard and applying a domain-based diagnostic framework. The review was conducted in accordance with PRISMA 2020 reporting standards [15].

## 2. Materials and Methods

This systematic review was conducted in accordance with the Preferred Reporting Items for Systematic Reviews and Meta-Analyses (PRISMA 2020) guidelines. The PRISMA Abstract Checklist and PRISMA Checklist are provided in the Supplementary Materials. This review was designed as an exploratory synthesis integrating evidence on diagnostic performance and morphologic associations of ultrasonographic upper airway parameters in adults with suspected obstructive sleep apnea (OSA).

### 2.1. Search Strategy

A systematic literature search was conducted in three electronic databases: PubMed, Scopus, and ScienceDirect. All databases were searched from inception to 20 February 2026. No restriction on year of publication was applied. Due to feasibility constraints, only English-language articles were included.

The search strategy combined controlled vocabulary (e.g., Medical Subject Headings [MeSH] in PubMed) with free-text terms related to obstructive sleep apnea, ultrasonography, and upper airway structures. Boolean operators (“AND”, “OR”) were used to combine search concepts. The core search structure was: (“obstructive sleep apnea” OR “OSA” OR “sleep apnea”) AND (“ultrasonography” OR “ultrasound” OR “sonography”) AND (“upper airway” OR “pharynx” OR “tongue” OR “lateral pharyngeal wall”).

Search syntax was adapted for each database according to its indexing system and search interface. Searches were performed in title, abstract, and keyword fields where applicable. After database searching, records were exported and deduplicated prior to title and abstract screening. The full database-specific search strategies are presented in Appendix A (Table A1).

### 2.2. Selection Criteria

Studies were included if they met the following criteria:

1. Enrolled adults (≥18 years);
2. Evaluated adults with suspected or clinically evaluated OSA, typically recruited from sleep clinics or PSG referral settings;
3. Used in-laboratory polysomnography (PSG) as the reference standard;
4. Assessed upper-airway structures using ultrasonography;
5. Reported one of the following:
  - Diagnostic performance metrics (e.g., sensitivity, specificity, area under the curve [AUC], cut-off values), or
  - Quantitative associations between ultrasonographic parameters and OSA severity (e.g., AHI correlation or group comparisons).

The primary diagnostic outcome was moderate-to-severe OSA (AHI ≥ 15 events/hour). When reported, additional AHI thresholds (e.g., ≥5 events/hour) were considered secondary outcomes.

Exclusion criteria were as follows:

1. Pediatric populations (<18 years);
2. Studies without PSG as the reference standard;
3. Interventional or treatment response studies;
4. Prognostic or surgical planning studies not focused on OSA diagnosis;
5. Non-upper airway imaging studies (e.g., MRI-only studies);
6. Review articles, editorials, conference abstracts, and non-English publications.

Study selection was performed in two stages: title and abstract screening followed by full-text assessment. Two reviewers independently screened all records, and discrepancies were resolved through discussion and consensus.

### 2.3. Data Extraction

Data extraction was performed independently by two reviewers using a standardized data collection form developed a priori. Extracted variables included: Study characteristics (author, year, country, study design, clinical setting), Participant characteristics (sample size, age, body mass index; BMI), Ultrasonographic parameters, Measurement protocol (static vs. dynamic assessment), AHI definition and PSG scoring criteria, Diagnostic performance metrics (cut-off values, sensitivity, specificity, area under the curve; AUC), Quantitative associations between ultrasonographic measurements and OSA severity.

Discrepancies between reviewers were resolved through discussion and consensus.

### 2.4. Risk of Bias Assessment

Methodological quality was independently assessed by two reviewers using the Quality Assessment of Diagnostic Accuracy Studies-2 (QUADAS-2) tool. The tool was adapted to accommodate studies reporting both diagnostic performance and morphologic associations. For morphologic association studies, the index test domain was interpreted in terms of measurement protocol standardization and blinding to PSG results. The following domains were evaluated: Patient selection, Index test (ultrasonography), Reference standard (polysomnography), Flow and timing.

Each domain was rated as low, high, or unclear risk of bias according to predefined signaling questions. Applicability concerns were also assessed for the domains of patient selection, index test, and reference standard. Disagreements between reviewers were resolved through discussion and consensus.

### 2.5. Data Synthesis

Given the heterogeneity in ultrasonographic parameters, acquisition protocols, measurement conditions (static versus dynamic), and AHI thresholds across studies, quantitative meta-analysis was not performed. Instead, a structured qualitative synthesis was conducted. The scope of this review was intentionally restricted to upper airway ultrasonographic parameters directly related to airway collapsibility, excluding systemic or non-airway ultrasonographic markers, to maintain conceptual and pathophysiologic coherence.

Included studies were categorized into two principal groups:

1. Diagnostic performance studies, defined as studies reporting diagnostic accuracy metrics (sensitivity/specificity and/or AUC), with either predefined or ROC-derived thresholds;
2. Morphologic association studies, defined as studies reporting quantitative relationships between ultrasonographic parameters and OSA severity (e.g., group comparisons or correlations with AHI) without validated diagnostic thresholds.

In addition to this classification, ultrasonographic parameters were further interpreted within three conceptual domains: dynamic airway behavior, tongue-related morphologic and tissue properties, and lateral pharyngeal wall and neck soft tissue measurements.

Findings were summarized narratively and tabulated according to study characteristics, diagnostic metrics, and risk of bias assessments. Risk of bias judgments were incorporated into the interpretation of diagnostic performance estimates.

## 3. Results

### 3.1. Study Selection

A total of 1050 records were identified through database searching (PubMed, n = 203, Scopus, n = 139, and ScienceDirect, n = 708). After removal of duplicates (n = 208), 842 records remained for title and abstract screening. Of these, 30 full-text articles were assessed for eligibility.

Twenty-four studies were excluded at the full text stage for the following reasons: did not report diagnostic performance metrics or quantitative morphologic associations with OSA severity (n = 11), interventional or treatment response design (n = 4), non–upper airway ultrasonography (n = 3), prognostic or surgical planning context (n = 4), or pediatric population (n = 2).

**Figure 1:**
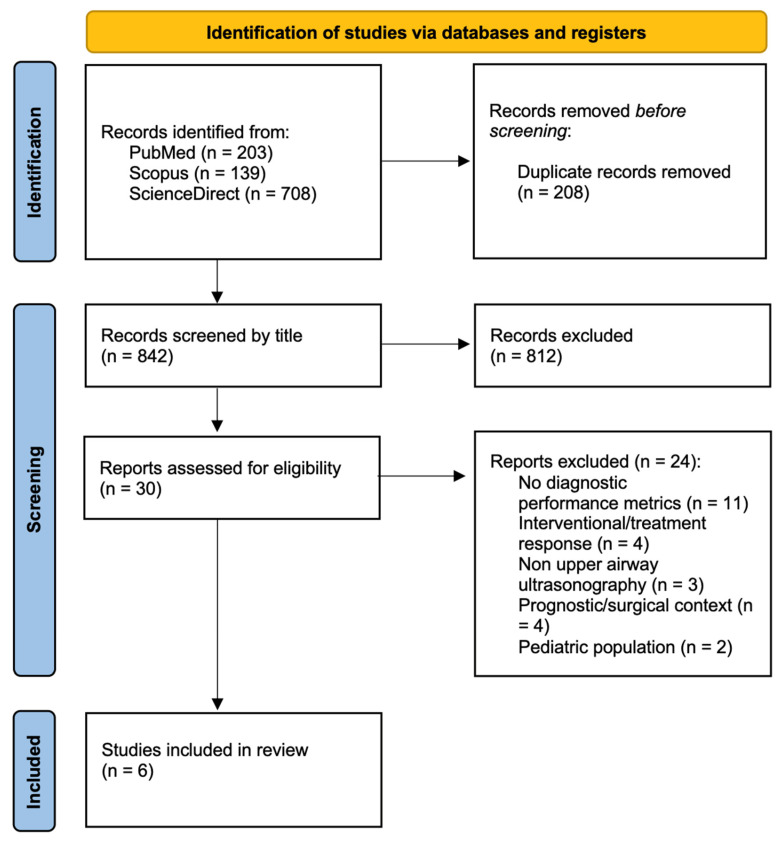
PRISMA 2020 flow diagram of the study selection process. A total of 1050 records were identified from database searches. After removal of 208 duplicates, 842 records were screened. Thirty reports were assessed for eligibility, and 24 were excluded for predefined reasons. Six studies were included in the qualitative synthesis.

Ultimately, six studies met the inclusion criteria and were included in the qualitative synthesis (Figure 1).

### 3.2. Study Characteristics

The characteristics of the included studies are summarized in Table 1. The six included studies were published between 2009 and 2025 and were conducted in tertiary sleep clinics or hospital-based settings. Sample sizes ranged from 40 to 208 participants, with most studies enrolling symptomatic individuals referred for polysomnography.

All studies used in-laboratory polysomnography (PSG) as the reference standard. Most studies defined OSA as an apnea–hypopnea index (AHI) ≥ 5 events/hour, whereas Lahav et al. [7] evaluated diagnostic performance primarily for moderate-to-severe OSA (AHI ≥ 15 events/hour).

The ultrasonographic parameters evaluated varied substantially across studies and included tongue base thickness, distance between lingual arteries, lateral pharyngeal wall thickness, dynamic retropalatal or retroglossal airway changes during inspiration or Müller maneuver, as well as oropharyngeal airway dimensional changes during deep inspiration and expiration, and tongue stiffness measured by shear-wave elastography. Both static and dynamic ultrasound protocols were employed, with dynamic maneuvers generally intended to simulate airway collapsibility.

### 3.3. Ultrasonographic Parameters Identified

Across the six studies, ultrasonographic assessment targeted structures implicated in upper airway obstruction. These parameters can be broadly categorized into:

1. Tongue-related measurements (e.g., tongue base thickness, shear-wave stiffness, lingual artery distance);
2. Lateral pharyngeal wall thickness;
3. Dynamic airway dimensional changes during deep inspiration/expiration or Müller maneuvers.

### 3.4. Diagnostic Performance Studies

Diagnostic performance metrics are summarized in Table 2. Three studies reported threshold-based diagnostic performance (cut-off with sensitivity/specificity and AUC), although only one study provided a full 2 × 2 contingency table with a prespecified threshold.

Lahav et al. (2009) [7] evaluated the distance between lingual arteries and reported a predefined cut-off (>30 mm), yielding a sensitivity of 80% and specificity of 67% for detecting moderate to severe OSA (AHI ≥ 15). This study provided a contingency table, enabling direct assessment of diagnostic accuracy.

Govindagoudar et al. (2023) [8] reported ROC-based analyses of dynamic retropalatal and retroglossal percentage changes. Retropalatal percentage change during inspiration demonstrated the highest performance (AUC 0.989; sensitivity 97%; specificity 93.3%), with similarly high values observed during Müller maneuver. These findings suggest that dynamic airway collapsibility parameters may offer strong discriminatory ability in selected clinical populations.

The shear-wave elastography study (Chang et al., 2020) [10] reported ROC-derived cut-off values for tongue stiffness parameters, with posterior tongue stiffness during Müller maneuver achieving an AUC of 0.88. However, not all elastographic parameters were reported with complete AUC values.

### 3.5. Morphologic Association Studies

Several studies reported significant quantitative associations between ultrasonographic parameters and OSA severity but did not provide full diagnostic contingency data (Table 3).

Chen et al. (2014) [11] demonstrated increased tongue base thickness during the Müller maneuver in OSA patients compared with controls, indicating structural enlargement associated with disease presence. Liu et al. (2025) [9] reported altered dynamic airway dimensions during deep inspiration, characterized by reduced lateral airway expansion and changes in airway configuration (AP/LAT ratio) in patients with OSA compared with controls.

Similarly, studies evaluating neck ultrasonography and lateral pharyngeal wall thickness reported significant correlations between soft tissue thickness and AHI. The neck ultrasonography study (Lal et al., 2023) [12] further reported a multivariable logistic regression model incorporating neck circumference, tongue base thickness, and lateral pharyngeal wall thickness. This model predicted severe OSA with a sensitivity of 72% and specificity of 76% in the derivation cohort; however, diagnostic performance was derived from a combined prediction model rather than from a single ultrasonographic parameter.

In addition, the shear-wave elastography study (Chang et al., 2020) [10] reported increased tongue stiffness during normal breathing (SWnb, middle tongue region) in OSA patients compared with controls, indicating altered biomechanical properties of the tongue in OSA.

Although these findings support the structural and pathophysiologic relevance of ultrasonographic measurements, the absence of standardized cut-off values or externally validated diagnostic thresholds limits their immediate clinical applicability for screening purposes.

### 3.6. Risk of Bias Assessment

The methodological quality of included studies, assessed using QUADAS-2, is presented in Table 4. Overall, the risk of bias was moderate across most studies.

Most studies demonstrated a moderate risk of bias, primarily related to patient selection and index test reporting. Several studies employed case–control designs or recruited participants from sleep clinics with a high pre-test probability of OSA, which may introduce spectrum bias. In addition, blinding procedures for ultrasonographic measurements were frequently not reported, resulting in an unclear risk in the index test domain. In contrast, the reference standard domain was consistently rated as low risk across all studies because in-laboratory polysomnography was used as the diagnostic reference. Flow and timing were generally appropriate, with ultrasound examinations performed within clinically relevant intervals relative to polysomnography.

## 4. Discussion

### 4.1. Principal Findings

This systematic review demonstrates that ultrasonographic upper-airway parameters show consistent associations with OSA severity; however, robust evidence of diagnostic accuracy with validated thresholds remains limited. Only a minority of studies reported predefined diagnostic thresholds with corresponding sensitivity, specificity, and AUC values aligned to clinically meaningful AHI thresholds. This diagnostic gap has also been highlighted in prior ultrasound-focused syntheses [5,6]. Unlike recent quantitative syntheses that pooled structural differences between OSA and non-OSA groups [6], the present review specifically evaluated predefined diagnostic thresholds within a strict PSG-based diagnostic framework.

Dynamic retropalatal measurements demonstrated the highest reported discriminatory performance in selected cohorts [8]. However, the predominance of populations with a high pre-test probability raises concerns regarding spectrum bias and potential overestimation of diagnostic accuracy [13]. In particular, the exceptionally high AUC reported by Govindagoudar et al. [8] should be interpreted cautiously, as small sample size and highly selected sleep-clinic populations may have inflated performance estimates and may limit generalizability to broader screening populations.

The limited number of eligible studies reflects strict methodological inclusion criteria, particularly the requirement for PSG as the reference standard and predefined diagnostic thresholds rather than an absence of exploratory ultrasonographic research.

### 4.2. Interpretation of Ultrasonographic Parameters

#### 4.2.1. Dynamic Airway Behavior

Dynamic parameters capture functional airway instability, which is central to the pathophysiology of OSA [2]. Measurements obtained during inspiratory or Müller maneuvers demonstrated high discriminatory performance in selected sleep clinic populations [8]. Deep-breath–related airway dimensional changes were also associated with OSA, supporting the relevance of dynamic airway geometry beyond retropalatal or retroglossal measurements [9]. However, the lack of standardized acquisition protocols limits reproducibility across centers [5].

#### 4.2.2. Tongue-Related Parameters and Tissue Properties

Tongue-related parameters, including tongue base thickness, lingual artery distance, and shear-wave elastography–derived stiffness, were consistently associated with OSA severity [7,10,11,12]. Increased tongue volume and altered biomechanical properties contribute directly to airway narrowing and collapse [2]. Nevertheless, externally validated diagnostic thresholds remain lacking.

#### 4.2.3. Lateral Pharyngeal Wall and Neck Soft Tissue

Lateral pharyngeal wall thickness and neck soft tissue parameters were associated with OSA severity in the included studies [12]. The lateral wall plays a recognized role in airway collapse [2]. However, validated cut-off values and standardized measurement planes are not yet established.

### 4.3. Methodological Considerations

Risk of bias was predominantly moderate across studies. Patient selection bias was common due to non-consecutive recruitment and case–control designs. Diagnostic accuracy literature has demonstrated that such designs may inflate performance estimates [13]. While PSG was consistently used as the reference standard [3], few studies reported complete 2 × 2 contingency data within a classical diagnostic accuracy framework. The observed heterogeneity across studies likely reflects differences in ultrasound acquisition protocols, anatomical measurement landmarks, and variability in AHI thresholds used to define OSA severity.

## 5. Clinical Implications

Ultrasonographic upper-airway assessment offers practical advantages, including accessibility, absence of ionizing radiation, and potential chairside integration. However, current evidence does not support its use as a standalone screening modality. Importantly, the role of ultrasonography may differ between screening for any OSA (AHI ≥ 5 events/hour) and identifying moderate-to-severe OSA (AHI ≥ 15 events/hour). Current evidence appears stronger for ultrasound as an adjunctive screening tool, whereas validated thresholds for diagnosing clinically significant OSA remain limited.

Within orthodontic and craniofacial practice, ultrasound may serve as an adjunctive tool for airway evaluation, complementing structural imaging and clinical risk assessment. Its role may be to refine pre-test probability in patients with craniofacial risk factors, rather than replace established diagnostic pathways such as PSG [3] or validated screening questionnaires including STOP-Bang [14]. Ultrasound may therefore be positioned as a preliminary risk-stratification tool in high-risk clinical populations rather than as a definitive diagnostic test.

## 6. Limitations and Future Research Directions

This review is limited by the small number of available studies and substantial heterogeneity in ultrasonographic techniques, parameter definitions, acquisition protocols, OSA definitions, and outcome thresholds. Additionally, several included studies employed case–control designs and highly selected sleep-clinic populations, which may overestimate diagnostic performance due to spectrum bias. Quantitative meta-analysis was not feasible because of methodological variability. In addition, the absence of prospective protocol registration may represent a limitation regarding transparency. The literature search was restricted to three electronic databases and English-language publications, which may have introduced language and selection bias and could have resulted in omission of relevant studies published in other languages. Formal certainty-of-evidence assessment was not performed because of substantial methodological and clinical heterogeneity across included studies. Furthermore, only one included study provided a complete diagnostic contingency table with a predefined threshold and externally validated diagnostic cut-off values remain limited.

Future studies should prioritize prospective consecutive suspected-OSA cohorts, predefined AHI thresholds (≥15 events/hour), standardized acquisition protocols, double-blinded assessment in which sonographers are unaware of PSG results and PSG scorers are unaware of ultrasound findings and reporting consistent with QUADAS-2 and PRISMA standards [15].

## 7. Conclusions

Ultrasonographic upper-airway parameters demonstrate promising morphologic and dynamic associations with OSA; however, their diagnostic utility remains limited. Dynamic measurements, tongue-related tissue properties, and lateral pharyngeal wall thickness each reflect distinct but complementary components of airway vulnerability. Nevertheless, high-quality diagnostic accuracy studies remain scarce, and standardized and externally validated thresholds are lacking. Ultrasonographic upper-airway assessment should currently be considered an adjunctive rather than a standalone screening approach. Rigorous prospective studies and external threshold validation are required before routine implementation in sleep medicine or orthodontic screening pathways can be recommended.

## Figures and Tables

**Table 1:** Study Characteristics.

Study	Country	Setting	Study Design	Sample Size	Mean Age (Years)	Mean BMI (kg/m^2^)	Ultrasound Parameters	AHI
Lahav et al., 2009 [7]	Israel	Sleep clinic	Prospective	41 adults with suspected OSA	49 ± 10	29.6 ± 4.3	Distance between lingual arteries (DLA)	≥5
Govindagoudar et al., 2023 [8]	India	Tertiary sleep clinic	Prospective	63 (33 OSA/30 overweight non-OSA)	44.8 ± 11.2	29.1 ± 3.8	Retropalatal % change, Retroglossal% change (inspiration and Müller maneuver)	≥5
Liu et al., 2025 [9]	China	Hospital-based	Case–control	208 (104 OSA/104 control)	47.2 ± 9.5	28.4 ± 3.6	AP and lateral airway diameter during deep breath	≥5
Chen et al., 2014 [11]	Taiwan	Sleep clinic	Case–control	40 (20 OSA/20 control)	42.7 ± 8.9	27.8 ± 2.9	Tongue base thickness (Müller maneuver)	≥5
Chang et al., 2020 [10]	Taiwan	Tertiary hospital	Prospective	46 (26 OSA/20 control)	46.3 ± 10.4	28.9 ± 3.2	Tongue stiffness (kPa)	≥5
Lal et al., 2023 [12]	India	Tertiary care center	Prospective	75 (50 OSA/25 control)	43.9 ± 11.4	34.5 ± 7.5	Tongue base thickness/Lateral pharyngeal wall thickness	≥5

All studies used in-laboratory polysomnography (PSG) as reference standard, all studies enrolled suspected OSA not general population, most defined OSA as AHI ≥ 5 events/hour.

**Table 2:** Ultrasonographic parameters and their association with obstructive sleep apnea. (Diagnostic Performance Studies).

Study	Ultrasound Parameter	Cut-Off	Sensitivity (%)	Specificity (%)	AUC	AHI Threshold
Lahav et al., 2009 [7]	Distance between lingual arteries (DLA)	>30 mm	80	67	-	≥15
Govindagoudar et al., 2023 [8]	Retropalatal % change (inspiration)	14.2%	97.0	93.3	0.989	≥5
	Retropalatal % change (Müller)	23.7%	97.0	100	0.988	≥5
	Retroglossal % change (inspiration)	13.4%	66.7	76.7	0.757	≥5
	Retroglossal % change (Müller)	28.7%	72.7	100	0.875	≥5
Chang et al., 2020 (SWE) [10]	SWmm (posterior third, Müller)	35.2 kPa	76.9	95.0	0.88	≥5
	SWmm (whole tongue, Müller)	27.6 kPa	69.2	85.0	0.82	≥5

SWE, shear wave elastography; SWmm, shear wave elastography during Müller maneuver.

**Table 3:** Ultrasonographic parameters and their association with obstructive sleep apnea. (Morphologic Association Studies).

Study	Ultrasound Parameter	Main Finding	Statistical Association
Chen et al., 2014 [11]	Tongue base thickness (Müller maneuver)	Increased thickness in OSA vs. controls	Significant group difference (*p* < 0.05)
Liu et al., 2025 [9]	Dynamic airway diameters (AP and lateral) and AP/LAT ratio	Reduced lateral airway expansion and altered airway configuration during deep inspiration in OSA	Significant difference between groups (*p* < 0.05)
Lal et al., 2023 [12]	Tongue base thickness/Lateral pharyngeal wall thickness	Increased thickness associated with OSA severity	Correlated with AHI
Chang et al., 2020 (SWE—additional parameters) [10]	SWnb (middle)	Higher stiffness in OSA	Significant difference (*p* < 0.01)

SWE, shear wave elastography; SWnb, shear modulus of the tongue during normal breathing.

**Table 4:** Risk of Bias Assessment using QUADAS-2.

Study	Patient Selection	Index Test	Reference Standard	Flow and Timing	Overall Risk
Lahav et al., 2009 [7]	🟡 Unclear	🟢 Low	🟢 Low	🟢 Low	🟡 Moderate
Govindagoudar et al., 2023 [8]	🟡 Unclear	🟢 Low	🟢 Low	🟢 Low	🟡 Moderate
Chang et al., 2020 [10]	🟡 Unclear	🟡 Unclear	🟢 Low	🟢 Low	🟡 Moderate
Chen et al., 2014 [11]	🟡 Unclear	🟡 Unclear	🟢 Low	🟢 Low	🟡 Moderate
Liu et al., 2025 [9]	🔴 High	🟡 Unclear	🟢 Low	🟢 Low	🟡 Moderate to high
Lal et al., 2023 [12]	🔴 High	🟡 Unclear	🟢 Low	🟡 Unclear	🔴 High

## Data Availability

Data is contained within the article.

## Supplementary Materials

The following supporting information can be downloaded at: https://www.mdpi.com/article/10.3390/jcm15103720/s1, File S1: PRISMA Abstract Checklist; File S2: PRISMA Checklist.

## Appendix A

**Table A1:** Full Search Strategy.

Database	Search Term
PubMed	(“Sleep Apnea, Obstructive”[MeSH] OR “obstructive sleep apnea”[tiab] OR “obstructive sleep apnoea”[tiab] OR OSA[tiab] OR OSAS[tiab]) AND (“Ultrasonography”[MeSH] OR ultrasound[tiab] OR ultrasonograph*[tiab] OR sonograph*[tiab]) AND (“upper airway”[tiab] OR airway[tiab] OR pharyn*[tiab] OR oropharyn*[tiab] OR hypopharyn*[tiab] OR tongue[tiab] OR lingual[tiab] OR “tongue base”[tiab] OR hyoid[tiab] OR submental[tiab] OR neck[tiab] OR “lateral pharyngeal wall”[tiab] OR parapharyn*[tiab] OR retroglossal[tiab] OR retropalatal[tiab]) AND (“Adult”[MeSH] OR adult*[tiab]) NOT (“Child”[MeSH] OR pediatric*[tiab] OR paediatric*[tiab])
Scopus	TITLE-ABS-KEY( (“obstructive sleep apnea” OR “obstructive sleep apnoea” OR OSA OR OSAS) AND (ultrasound OR ultrasonograph* OR sonograph* OR “ultrasonic”) AND (“upper airway” OR airway OR pharyn* OR oropharyn* OR hypopharyn* OR “soft palate” OR tongue OR lingual OR “tongue base” OR hyoid OR submental OR “sub-mental” OR “lateral pharyngeal wall” OR parapharyn* OR retroglossal OR retropalatal) ) AND TITLE-ABS-KEY(adult OR adults) AND NOT TITLE-ABS-KEY(pediatric* OR paediatric* OR child*)
ScienceDirect	(“obstructive sleep apnea” OR OSA) AND (ultrasound OR ultrasonography OR sonography) AND (airway OR “upper airway” OR pharynx OR tongue) AND adult
